# Survey of Toxin–Antitoxin Systems in *Erwinia amylovora* Reveals Insights into Diversity and Functional Specificity

**DOI:** 10.3390/toxins11040206

**Published:** 2019-04-06

**Authors:** Teja Shidore, Quan Zeng, Lindsay R. Triplett

**Affiliations:** Department of Plant Pathology and Ecology, the Connecticut Agricultural Experiment Station, New Haven, CT 06511, USA; teja.shidore@ct.gov (T.S.); Quan.Zeng@ct.gov (Q.Z.)

**Keywords:** toxin–antitoxin system, *Erwinia amylovora*, YeeV, CbtA, ParE, Doc, cell elongation

## Abstract

Toxin–antitoxin (TA) systems are diverse genetic modules with demonstrated roles in plasmid stability, stress management, biofilm formation and antibiotic persistence. However, relatively little is known about their functional significance in plant pathogens. In this study we characterize type II and IV TA systems in the economically important plant pathogen *Erwinia amylovora*. Hidden Markov Model (HMM) and BLAST-based programs were used to predict the identity and distribution of putative TA systems among sequenced genomes of *E. amylovora* and other plant-associated *Erwinia* spp. Of six conserved TA systems tested for function from *E. amylovora*, three (CbtA/CbeA, ParE/RHH and Doc/PhD) were validated as functional. CbtA was toxic to *E. amylovora*, but not to *Escherichia coli*. While the *E. coli* homolog of CbtA elicits the formation of lemon-shaped cells upon overexpression and targets cytoskeletal proteins FtsZ and MreB, *E. amylovora* CbtA led to cell elongation and did not interact with these cytoskeletal proteins. Phylogenetic analysis revealed that *E. amylovora* CbtA belongs to a distinct clade from the CbtA of pathogenic *E. coli*. This study expands the repertoire of experimentally validated TA systems in plant pathogenic bacteria, and suggests that the *E. amylovora* homolog of CbtA is functionally distinct from that of *E. coli*.

## 1. Introduction

In recent decades, numerous toxin–antitoxin (TA) systems have been identified and implicated in diverse regulatory and fitness roles in model bacterial species and mammalian pathogens. In contrast, relatively little is known about the diversity and function of TA systems in plant-associated bacteria, including agricultural pathogens, symbionts and biocontrol organisms. A small handful of functional studies have suggested that TA systems are relevant to crop disease, with roles in plasmid maintenance, stress responses, biofilm formation or virulence in diverse plant pathogenic bacteria (reviewed in [1]). A better understanding of TA system composition and roles in plant-associated bacteria could open up new avenues for understanding environmental fitness and stress persistence in these organisms, and potentially for improving the effectiveness of disease control and agricultural microbial products.

The plant pathogen *Erwinia amylovora* causes fire blight, an economically important disease of apple and pear that poses a significant threat to rosaceous fruit tree cultivation globally [2]. *E. amylovora* is divided into two genetically homogeneous groups based on host specificity: Spiraeoideae-infecting (SI) strains can cause disease on apple and pear, while *Rubus*-infecting strains are limited to bramble hosts [3]. Although no TA systems have previously been studied in *E. amylovora*, it is a species with well-characterized virulence mechanisms, ample genomic resources, standardized protocols for genetic manipulation and close genetic relatedness to the enteric human pathogens that have served as models for TA system function. *E. amylovora* also faces a diverse set of abiotic and biotic stressors, as the disease cycle encompasses both epiphytic and endophytic stages of growth. Epiphytic *E. amylovora* populations undergo periods of rapid growth while encountering antibiotic and copper disease control sprays as well as antagonistic bacteria and phages in the floral microbiome, while endophytic populations are challenged by host resistance and temperature fluctuations [2,4]. *E. amylovora* overwinters in cankers on infected branches and is thought to enter a dormant Viable But Not Culturable state during this time [5].

Because of its genetic tractability and the widespread reliance on bactericidal sprays for its control, *E. amylovora* presents a promising model system to advance the understanding of the role of TA systems in plant pathogen lifestyles. This work represents the first steps in this area of study, reporting a genomic and functional survey of *E. amylovora* TA systems. Genomic prediction programs were used to identify the presence and distribution of type II and IV TA systems in diverse pathogenic and nonpathogenic *Erwinia* species; six predicted TA systems were selected for validation. Ectopic expression studies confirmed the TA system function of three candidates and showed mild growth-suppression activity of a fourth predicted gene. Phenotypic differences were observed upon expression of the *E. amylovora* type IV toxin CbtA and its *E. coli* homolog, suggesting distinct functions or targets for the two homologs. These findings broaden the known repertoire of functional TA systems in plant pathogens and establish groundwork for future studies of TA systems in *E. amylovora*.

## 2. Results

### 2.1. Identity and Distribution of Type II and IV TA Systems in Pathogenic and Nonpathogenic Erwinia spp.

We selected *E. amylovora* strain CTBT3-1, a sequenced high-virulence strain [3], to perform functional validation studies of protein–protein TA systems. While most known roles of TA systems have been attributed to those of type II, the single type IV family identified has a similar operon structure and is incorporated into the training sets of multiple TA system prediction programs [6,7]. Thus, we included both type II and IV systems in the study of the occurrence, abundance and specificity of TA systems in *E. amylovora*. TA system prediction was first performed in both the *E. amylovora* reference genome CFBP1430 and in the draft CTBT3-1 genome using TAfinder, a web-based annotation-dependent program that combines protein BLAST and HMMer3-based predictions [6]. TAfinder predicted nine candidate chromosomal and plasmid-encoded TA systems in both strains, encoding putative toxin inhibitors of translation (VapC, Doc and HicA, GNAT domain), replication (ParE), cell division (YeeV/CbtA, henceforth CbtA) and proteins of unknown function (COG3916 proteins) (Table 1). BLASTP search analysis indicated that the predicted TA system with COG3916 and Pfam 00196 domain proteins was likely a quorum-sensing system; this system was not studied further.

To validate the prediction results of TAfinder, we performed another prediction using SLING, in which toxins were predicted based on a curated Hidden Markov Model (HMM) file followed by the identification of linked genes and BLAST clustering to identify clusters of TA system homologs (>30% identity) [7]. Prediction results by SLING largely agree with those of TAfinder; out of the seven total TA systems predicted in CTBT3-1 by SLING, five of them were the same ones predicted by TAfinder; two additional modules (DUF4258-domain and polyketide cyclase domain toxins, i.e., RatA) were uniquely predicted by SLING and two modules (Xre/GNAT and ParE/RHH) predicted by TAfinder were not identified by SLING (Table 1).

**Figure 1:**
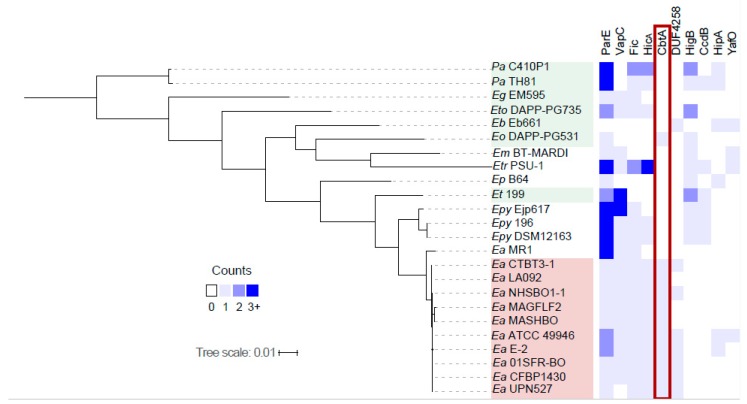
Identification and distribution of type II and IV TA systems. Representative toxins of the predicted TA systems are depicted. Abbreviations used—*Pa*: *Pantoea agglomerans*; *Eg*: *Erwinia gerundensis*; *Eto*: *Erwinia toletana*; *Eb*: *Erwinia billingiae*; *Eo*: *Erwinia oleae*; *Em*: *Erwinia mallotivora*; *Etr*: *Erwinia tracheiphila*; *Ep*: *Erwinia persicina*; *Et*: *Erwinia tasmaniensis*; *Epy*: *Erwinia pyrifoliae*; *Ea*: *Erwinia amylovora.*

We hypothesized that TA systems with important roles in the *E. amylovora* disease cycle may be conserved among or specific to pathogenic *E. amylovora* strains relative to closely related nonpathogenic *Erwinia* species. To understand the distribution of TA systems in *Erwinia* species and to identify *E. amylovora*-specific TA systems, we expanded the TA system search across representative pathogenic and nonpathogenic *Erwinia* spp. using SLING. The presence and absence of TA systems in each genome was analyzed in relevance to their pathogen status and phylogeny based on a whole-genome phylogenetic analysis (Figure 1). The analysis included 10 *E. amylovora* genomes from the economically important Spiraeoideae-infecting (apple and pear-infecting) clade (Figure 1, highlighted in red) and one from the genetically distinct *Rubus*-infecting clade, in addition to five nonpathogenic *Erwinia* spp. (highlighted in green) and six other pathogenic *Erwinia* spp. Two strains of the nonpathogen *Pantoea agglomerans* were incorporated as outliers to root the tree.

SLING predicted TA systems representing 24 toxin families among the 24 genomes, with a range of six to 27 systems per genome (File S1). *E. amylovora* strains were predicted to harbor an average of 7.6 TA systems, lower than the average of 15.8 among the six genomes of other *Erwinia* pathogens (*P* = 0.0018) and 11.6 among the seven genomes of nonpathogenic *Erwinia* ssp (*P* = 0.018, Figure S1). Of the 13 toxin families detected among *E. amylovora* genomes, seven were conserved in all or nearly all of the strains, consistent with the relatively low genetic diversity of *E. amylovora* (File S1). Six toxin families were only detected in three or fewer strains (File S1); of these, YafO, PemK and HipA toxin genes were detected in strain ATCC 49946, suggestive of an association with the plasmid pEA72 [8]. The predicted repertoire of the *Rubus*-infecting MR1 strain also had several differences from other *Erwinia* strains (File S1, Figure 1). Five TA systems (ParE, VapC, Fic, HicA and CbtA) identified in CTBT3-1 by both TAFinder and SLING were conserved in all or all but one *E. amylovora* genome (Figure 1). The TA system most specific to *E. amylovora* was the type IV CbtA/CbeA module, predicted in all SI *E. amylovora* strains and only in one other genome (Figure 1). In summary, five candidate TA systems were predicted in CTBT3-1 by two programs, two were predicted uniquely by SLING and three were predicted uniquely by TAfinder. The five TA systems predicted by both programs were detected in all or almost all SI *E. amylovora* strains, of which the type IV system CbtA/CbeA was predominantly specific to *E. amylovora*.

### 2.2. Functional Validation of Predicted Toxins and TA Systems

**Figure 2:**
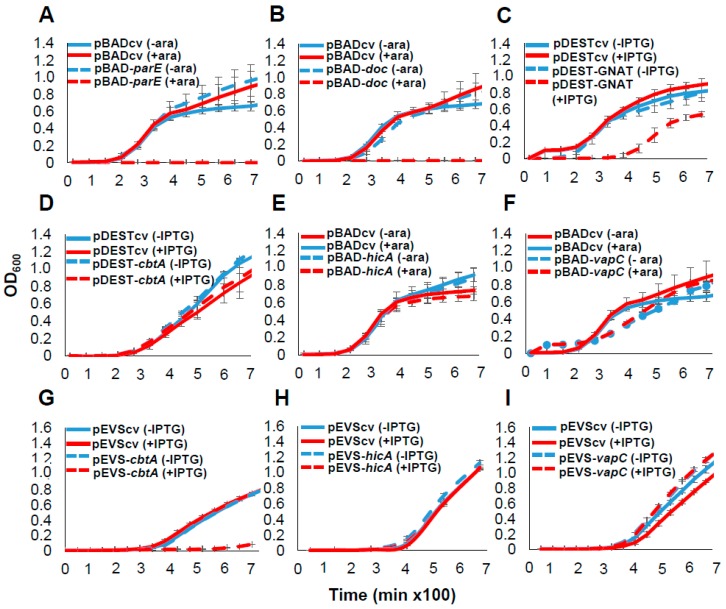
Effect of ectopic expression of predicted toxins on the growth of *E. coli* and *E. amylovora*. The growth of *E. coli* strain BL21 (DE3) harboring (**A**) pBAD-*parE*, (**B**) pBAD-*doc* (**C**) pDEST-GNAT (**D**) pDEST-*cbtA*, (**E**) pBAD-*hicA*, (**F**) pBAD-*vapC* and the growth of the *E. amylovora* strain CTBT3-1 harboring (**G**) pEVS-*cbtA*, (**H**) pEVS-*hicA*, (**I**) pEVS-*vapC* was monitored. Analysis of strains carrying pBAD33 plasmids was performed under arabinose inducible (+ara) or non-inducible (-ara) conditions while analysis of those carrying pDEST527 and pEVS143 plasmids was performed under IPTG inducible (+IPTG) or non-inducible conditions (-IPTG) of expression. Control vectors (cv) were included for every analysis. Bacterial growth is presented on a linear scale to allow visualization of moderate or mild effects of toxin expression on bacterial growth. Data points and error bars represent the means and standard deviations of three biological replicates.

We selected a subset of six *E. amylovora* TA systems for functional validation studies. These included the five TA systems that were identified by both programs and conserved in *E. amylovora* (CbtA/CbeA, ParE/RHH, Doc/PhD, HicA/HicB and VapC/PhD). In addition, we included the GNAT/Xre system predicted by TAfinder but not SLING, as this module shared clear sequence similarity to functionally characterized GNAT domain systems. First, predicted toxins were expressed in *E. coli* and the effect on bacterial growth was monitored. The *cbtA* and *GNAT* toxin genes were cloned into an IPTG (isopropyl-ß-D-thiogalactopyranoside)-inducible vector, and the *parE*, *doc*, *hicA* and *vapC* genes were cloned into a vector with arabinose-inducible pBAD promoter due to difficulty cloning some toxins into the IPTG-inducible vector. The expression of *parE* and *doc* caused drastic reductions in the growth of *E. coli*, whereas the expression of the gene encoding the GNAT domain protein led to a mild suppression of growth (Figure 2A–C). The induction of cloned genes *cbtA*, *hicA* or *vapC* did not have a growth suppressive effect in *E. coli* (Figure 2D–F). To determine whether some genes could be toxic in a species-specific manner, we next cloned *cbtA*, *hicA* and *vapC* in the *E. amylovora* strain CTBT3-1 under the IPTG-inducible pEVS promoter. A drastic reduction in apparent *E. amylovora* growth was observed upon *cbtA* expression (Figure 2G). Induction of the cloned *hicA* and *vapC* genes did not affect the growth of *E. amylovora* (Figure 2H,I).

**Figure 3:**
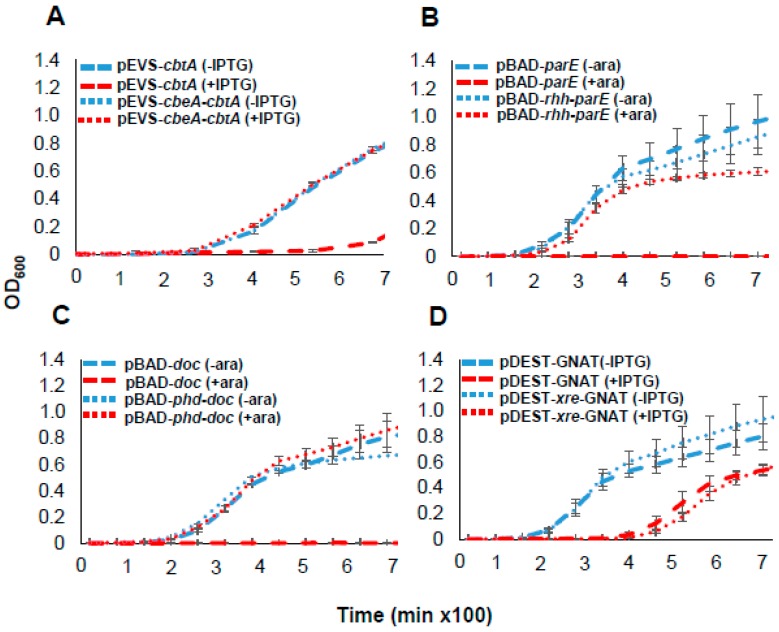
Effect of co-expression of putative antitoxins with its cognate toxin on the growth of *E. coli* or *E. amylovora*. The growth of *E. amylovora* strain CTBT3-1 harboring (**A**) pEVS-*cbtA* or pEVS-*cbeA-cbtA*; *E. coli* strain BL21 (DE3) harboring (**B**) pBAD-*parE* or pBAD-*rhh-parE*, (**C**) pBAD-*doc* or pBAD-*phd*-*doc*, (**D**) pDEST-GNAT or pDEST-*xre*-GNAT was monitored. Analysis of strains carrying pDEST527 and pEVS143 plasmids was performed under IPTG inducible (+IPTG) or non-inducible conditions (-IPTG) while analysis of those carrying pBAD33 plasmids was performed under arabinose inducible (+ara) or non-inducible (-ara) conditions of expression. Bacterial growth is presented on a linear scale to allow visualization of moderate or mild effects of toxin expression on bacterial growth. Data points and error bars represent the means and standard deviations of three biological replicates.

To test whether each toxin functioned as part of a complete toxin–antitoxin system, we co-expressed functional toxins with predicted cognate antitoxins. Growth suppressive effects of CbtA in *E. amylovora*, and of ParE and Doc in *E. coli*, were antagonized by the co-expression of their respective antitoxins (Figure 3A–C). However, a clone harboring the entire *xre-GNAT* module did not relieve the mild growth suppression caused by the GNAT toxin (Figure 3D). These results demonstrate that *E. amylovora* CbtA, ParE, GNAT and Doc proteins exhibit growth suppressive phenotypes, and that CbtA/CbeA, ParE/RHH and Doc/PhD form functional TA systems. Of these, CbtA toxic function was specific to *E. amylovora* and not observed in *E. coli*.

### 2.3. CbtA Toxin from CTBT3-1 Causes Cell Elongation That Is Suppressed by CbeA

**Figure 4:**
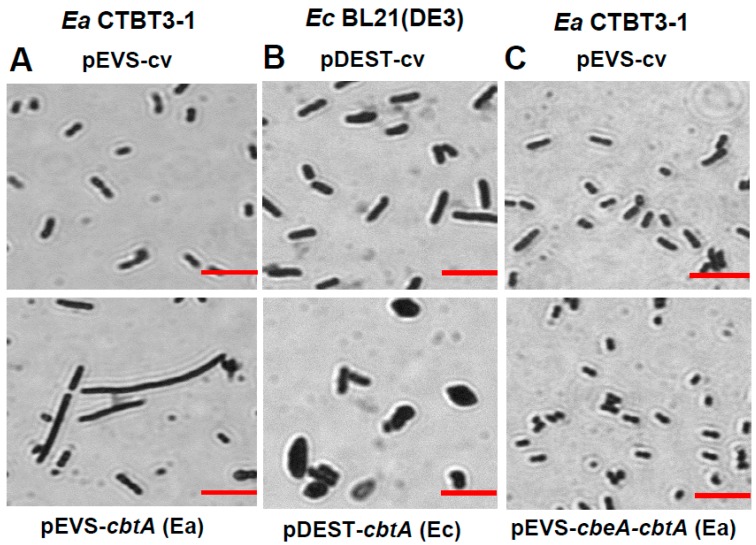
Effect of ectopic expression of toxin CbtA and co-expression of antitoxin CbeA on cell morphology. Overnight cultures of *E. amylovora* strain CTBT3-1 transformed with (**A**) pEVS-cv or pEVS-*cbtA* (Ea), (**B**) *E. coli* strain BL21 transformed with pDEST-cv or pDEST-*cbtA* (Ec) and (**C**) *E. amylovora* strain CTBT3-1 transformed with pEVS-cv or pEVS-c*beA-cbtA* (Ea) were diluted and grown under IPTG induction. Induction was performed at OD_600_ ~0.3. Cells were harvested 5 h post induction, heat fixed and stained with crystal violet. Images were captured using a Zeiss Axio Microscope. Bar represents 10 µm.

We demonstrated that CbtA/CbeA from strain CTBT3-1 forms a functional TA system, is only present in SI *E. amylovora* but not in most other *Erwinia* species and exhibits toxicity specific to *E. amylovora*. The species-specific function and conservation of CbtA/CbeA among SI *E. amylovora* made it an interesting candidate for further investigation. Ectopic expression of a prophage-encoded CbtA and its homologs YkfI and YpjF from *E. coli* were previously reported to inhibit both cell elongation and lateral expansion, triggering the formation of lemon-shaped cells [9,10,11]. In this study, we wanted to determine whether the *E. amylovora* homolog, henceforth CbtA (Ea), has a similar effect on cell morphology. Upon the overexpression of CbtA (Ea) in its native strain CTBT3-1, the cells appeared elongated (Figure 4A), but not lemon-shaped cells as was observed upon the expression of CbtA (Ec) in *E. coli* (Figure 4B) and as previously reported [9,10]. Co-expression of CbtA (Ea) with the CbeA (Ea) antitoxin abrogated the morphological alteration caused by the toxin, confirming its antagonistic function (Figure 4C). This suggests that CbtA (Ea) inhibits cell division but not lateral expansion in *E. amylovora*, and represents a functional difference to CbtA homologs from *E. coli*.

### 2.4. CbtA (Ea) Is Part of a Separate Clade from CbtA of Pathogenic E. coli

**Figure 5:**
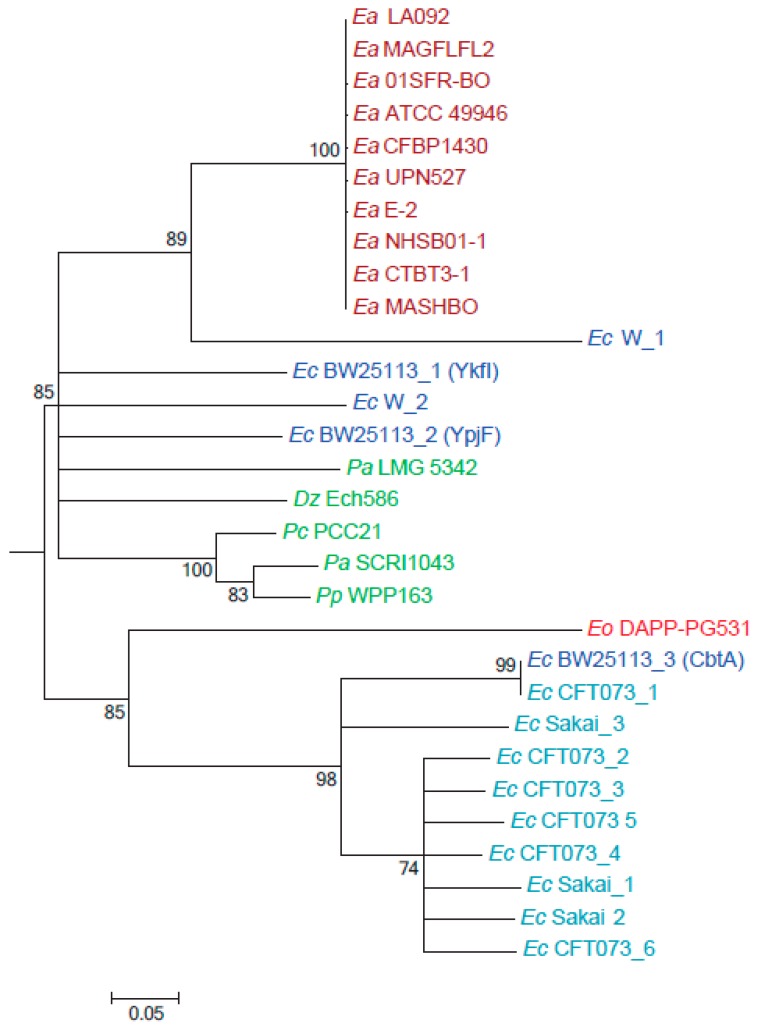
Phylogeny of CbtA homologs from *E. amylovora*, *E. coli* and other phytopathogenic strains. Neighbor joining tree of aligned amino acid sequences from 30 CbtA homologs. Sequence accessions are listed in Table S1. The datasets consisted of 46 amino acids after pairwise gap elimination. Bootstrap percentages for 1000 replicates are shown next to branches. Units represent the number of amino acid substitutions per site. Abbreviations used—*Ea*: *Erwinia amylovora*; *Ec*: *E. coli*; *Dz: Dickeya zeae*; *Pc*: *Pectobacterium carotovorum*; *Pa*: *Pantoea ananatis*; *Pp*: *Pectobacterium atrocepticum; Pp*: *Pectobacterium parmentieri; Eo*: *Erwinia oleae.*

Characterized *cbtA/cbeA*-like modules in *E. coli* are prophage-encoded [11,12], providing a mechanism for horizontal transfer between bacterial species and for copy number expansion within species. Unlike in *E. amylovora*, which encodes a single *cbtA* copy, these modules have expanded to a high number of copies in epidemic strains of human pathogenic *E. coli* (Figure 5), suggestive of a potential selective advantage in pathogen fitness [13,14]. Having shown that the expression of CbtA (Ea) has a different effect on cell morphology than CbtA (Ec), we hypothesized that CbtA homologs from plant pathogens might have a different origin from those in human pathogens. Taxon-specific BLAST searches against known phytopathogen genera determined that the *cbtA/cbeA* module was limited to plant pathogens of the family Enterobacteriaceae, with predicted single homologs in *Pantoea*, *Pectobacterium*, *Dickeya* and *Enterobacter* species. Phylogenetic analysis was performed on CbtA homologs from *Erwinia* and other plant pathogens, from two nonpathogenic *E. coli* strains, the K-12 derivative BW25113 and strain W, which respectively encode three and two homologs of CbtA. We also included two epidemic pathogen *E. coli* strains, the uropathogenic (ExPEC) strain CFT037 and the enterohemorrhagic (EHEC) strain O157:H7 str. Sakai, which respectively encode six and three CbtA homologs. Neighbor-joining phylogenetic analysis revealed that *E. amylovora* CbtA is part of a larger clade shared by CbtA from other plant pathogens, as well as four of five homologs from nonpathogenic *E. coli* including those previously characterized as YkfI and YpjF (Figure 5). Within this clade, *E. amylovora* CbtA shares a common ancestor with a homolog from *E. coli* strain W. CbtA copies from the two epidemic *E. coli* strains were all grouped into a separate clade with CbtA (Ec) from BW25113. Interestingly, CbtA from the olive knot-associated endophyte *E. oleae* also fell into this clade, indicating that the gene was acquired from a different source than other enteric plant pathogens. This analysis suggests that there are two divergent clades of CbtA, one of which is associated with plant pathogens and nonpathogenic *E. coli*, and one which has expanded to large numbers in epidemic *E. coli* strains. However, the two major clades of CbtA are not associated with different morphological functions, as BW25113 proteins YfkI and YpjF were previously found to induce lemon-shaped cells [9,11].

### 2.5. CbtA (Ea) Did Not Interact with Known Targets of CbtA (Ec) in a Yeast Two-Hybrid Study

Previous studies on CbtA (Ec) demonstrated that the toxin targets cytoskeletal proteins FtsZ and MreB, leading to the simultaneous inhibition of cell division and disruption of cell shape [9,10,15]. The antitoxin CbeA antagonizes toxin activity by binding directly to FtsZ and MreB and enhancing their bundling [15]. All three toxin homologs from *E. coli* interact with the H6/H7 loop of FtsZ, inhibiting cell division, and CbtA and YpjF interact with conserved residues in MreB. FtsZ and MreB proteins in *E. amylovora* are nearly identical to those in *E. coli* (FtsZ: 95% ID, MreB: 99% ID), including the total conservation of known CbtA-interacting residues. Although CbtA (Ea) shares only 63% sequence identity with CbtA (Ec), the CbtA (Ec) residues critical for FtsZ (F65) and MreB interaction (R15) in *E. coli* [9] are conserved in CbtA (Ea). We sought to determine whether CbtA (Ea) and CbeA (Ea) targeted the same cytoskeletal proteins as CbtA (Ec) and CbeA (Ec). Given that CbtA (Ea) appears to inhibit cell division but not lateral expansion in *E. amylovora*, we hypothesized that it would interact with FtzZ, but not with MreB.

**Figure 6:**
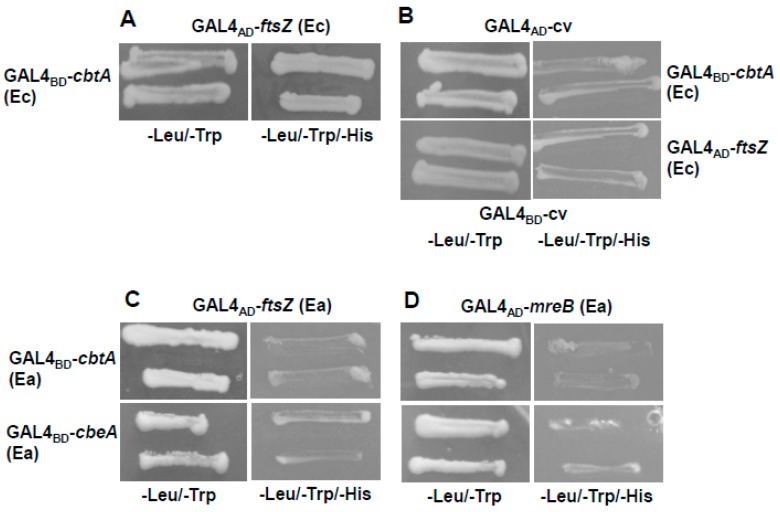
Assessment of CbtA and CbeA interacting proteins using a yeast two-hybrid (Y2H) assay. Growth of *Saccharomyces cerevisiae* strain Y2H gold co-transformed with (**A**) GAL4_BD_-*cbtA* (Ec) bait and GAL4_AD_-*ftsZA* (Ec) prey fusion construct; (**B**) GAL4_BD_-*cbtA* (Ec) bait and GAL4_AD_-cv prey fusion construct or GAL4_AD_-*ftsZA* (Ec) prey and GAL4_BD_-cv bait fusion construct; (**C**,**D**) GAL4_BD_-*cbtA* (Ea) or GAL4_BD_-*cbeA* (Ea) bait and GAL4_AD_-*ftsZ* (Ea) or GAL4_AD_-*mreB* (Ea) prey fusion construct was assessed on SD/−Leu/−Trp and SD/−Leu/-Trp/-His plates 3 days after transformation.

Yeast two-hybrid (Y2H) assays were performed to determine the interaction. We first determined if CbtA (Ec) and FtsZ (Ec) from strain BW25113 interacted in our assay system. As seen in Figure 6A, the co-transformation of *cbtA* (Ec) bait fusion construct and *ftsZ* (Ec) prey fusion construct lead to the growth of the transformant on selection plates, indicating that the interaction between the CbtA toxin and the cytoskeletal protein FtsZ from *E. coli* was reproducible in our system. No growth was observed after the co-transformation of *cbtA* (Ec) *or ftsZ* (Ec) constructs with empty vector controls (Figure 6B). On the co-transformation of the *cbtA* (Ea) bait and either of the *ftsZ* (Ea) or *mreB* (Ea) prey fusion constructs, no growth could be observed on selection plates (Figure 6C,D). A similar result was obtained when a bait-antitoxin fusion construct was co-transformed with prey fusion constructs of cytoskeletal proteins (Figure 6C,D). Co-transformations were also performed after interchanging the inserts of the bait and prey plasmids, but no interaction was observed. In summary, unlike their homologs in *E. coli*, CbtA (Ea) and CbeA (Ea) did not interact with the cytoskeletal proteins FtsZ and MreB from *E. amylovora*.

## 3. Discussion

TA systems are nearly ubiquitous in free-living bacteria and have emerged as important contributing factors to stress persistence and pathogen competence in model enteric pathogens. Plant pathogens share many of the virulence and regulatory strategies as mammalian pathogens and face many similar stress conditions, but little is known about the distribution or function of TA systems in these bacteria. In this study, dual prediction analyses identified 10 candidate type II and IV TA systems in the fire blight pathogen *E. amylovora* CTBT3-1, of which five were predicted by both SLING and TAfinder methods. Genomic distribution analysis showed that these five systems were highly conserved among isolates of the apple- and pear-infecting clade of *E. amylovora.* Ectopic expression studies of six selected systems confirmed the function of the CbtA/CbeA, ParE/RHH and Doc/PhD TA systems. A solo predicted GNAT-domain toxin also suppressed growth to a moderate degree upon overexpression. To our knowledge, this study presents the first distribution and functional analysis of TA systems in a plant pathogen of the family Enterobacteriaceae.

Comparative analysis demonstrated that, while most toxin families in *E. amylovora* were also found throughout other *Erwinia* species, the type IV CbtA/CbeA system was predominantly limited to *E. amylovora* species. This system was even absent from *E. pyrifoliae*, a closely related pathogen generally limited to Asian pear (*Pyrus pyrifolia*), and from the MR1 strain of *E. amylovora*, which is restricted to bramble hosts. This could suggest that CbtA (Ea) may have a role specific to the pathogenic lifestyle of Spiraeoideae-infecting *E. amylovora*. Wen and colleagues demonstrated that the deletion of the three toxin genes encoding CbtA, YkfI and YpjF from *E. coli* decreased resistance to oxidative stress, suggesting a function in stress survival. Deletion of the antitoxins contributed to biofilm formation [11]. Mutational studies are underway to determine whether CbtA (Ea) contributes to stress tolerance or pathogen fitness in *E. amylovora*.

Of the four toxin genes validated in this study, only CbtA (Ea) was toxic to *E. amylovora* and not to *E. coli*. Overexpression of putative TA system toxins from *S. pneumoniae* in its native strain and the heterologous host *E. coli* also led to divergent effects, illustrating the importance of functional TA system studies in the native strain background [16]. Host-specific toxicity of the CbtA family toxins was also previously observed [11]; this is consistent with the CbtA inhibition of a specific protein target, rather than a nucleic acid. CbtA (Ea) toxicity in *E. amylovora* was accompanied by the formation of elongated cells, not lemon-shaped cells as was induced by CbtA (Ec) in *E. coli*; this was suggestive of a sole interaction with the division protein FtsZ. However, we were unable to demonstrate an interaction between CbtA (Ea) or CbeA (Ea) and cytoskeletal proteins from *E. amylovora*. Many factors can interfere with interaction in yeast, so we cannot rule out CbtA (Ea) interaction with FtsZ or MreB. Given that CbtA (Ea) and CbtA (Ec) share only 63% identity and fall into separate clades, these findings raise the possibility that members of the CbtA (Ea) clade could have a distinct target in the organisms that express them. Were this the case, filamentation might indicate the inhibition of other cell division proteins, or of the induction of a general stress response. A high-throughput screening or co-immunoprecipitation approach may be needed to detect the target of CbtA in *E. amylovora.* The sequence and phenotypic divergence of CbtA (Ea) and CbtA (Ec) demonstrates the value of studying related TA systems in the context of diverse organisms.

The ParE toxin and Doc/Phd systems functionally validated in this study are from families that have been implicated in diverse functions. ParE toxins may contribute to plasmid stability and chromosomal DNA maintenance [17,18]. The ParE-like StbE*_pEP_*_36_ toxin of *E. pyrifoliae* also induces a viable but non-culturable cell stage [19]. The doc/phD module of *Salmonella* Typhimurium contributes to persister formation [20]. The predicted *vapC* and *hicA* genes did not cause growth suppression in either bacterial species in our study; however, in the absence of expression assays we cannot conclusively rule out the possibility of TA system function at these loci. Conversely, there is always the possibility that the overexpression of genes from strong promoters will cause phenotypes that may not be relevant under normal physiological conditions. This may apply in particular to the predicted GNAT-domain protein, which has a weak growth suppressive phenotype even under strong expression conditions. Deletion mutagenesis of TA loci will be necessary to identify any relevance they may have to pathogen fitness traits in *E. amylovora*. Although we limited this study to six TA systems, the four remaining predicted systems may also be functional; previous comparison of SLING and TAfinder demonstrated that the combination of these prediction methods may be complementary in identifying functional systems [7]. The validation of three TA systems conserved in *E. amylovora* will help perform future investigation on their roles in the disease cycle of fire blight and other plant diseases.

## 4. Materials and Methods

### 4.1. Prediction of TA Systems in Erwinia Species

The prediction of TA systems in *E. amylovora* was performed using the TAfinder web server with the RefSeq assembly file for experimental strain CTBT3-1 (accession ASM273220) as input, and using the chromosome and pEA29 plasmid assemblies for the reference strain, CFBP1430 (accessions NC_013961 and NC_013957, respectively). Default parameters were used. To perform SLING-based TA system prediction across multiple genomes, whole-genome FASTA and GFF assemblies of representative *Erwinia* and *Pantoea* species were downloaded from the NCBI assembly database (unique identifiers: 163858, 265038, 620271, 628598, 697808, 127921, 218861, 647371, 45148, 110288, 98128, 375358, 1455761, 1933091, 445178, 670758, 676418, 1419211, 1419231, 1419251, 1419271, 670778, 784721 and 2041111). SLING was run with default settings using the provided HMM profiles for toxins. Counts of individual TA systems were consolidated by toxin family for incorporation into the heatmap in Figure 1.

### 4.2. Phylogenetic Analyses

Whole-genome phylogenetic analysis was performed with GToTree [21] using the assembly FASTA files from Section 4.1 as input sequence, using default settings and HMM profiles for Gammaproteobacteria. The tree was drawn and annotated with toxin family counts using the Interactive Tree of Life server [22]. For phylogenetic analysis of CbtA homologs, homologs with >40% identity were considered for analysis. Accession numbers for the CbtA homologs are provided in Table S1. Sequence alignment (ClustalW) and neighbor-joining phylogenetic analysis was conducted in MEGA6 with the default settings. The tree was collapsed using TreeCollapserCL4 (http://emmahodcroft.com/TreeCollapseCL.html) with a threshold value of 0.7.

### 4.3. Strains, Plasmids and Culture Conditions

The bacterial strains and plasmids used in this study are listed in Table S2. *E*. *coli* strain DH5α was used for plasmid maintenance, strain BL21 (DE3) for growth curve and cell morphology analysis and *Erwinia amylovora* strain CTBT3-1 for growth curve and cell morphology analysis. *Saccharomyces cerevisiae* strain Y2H gold (TakaraBio USA, Mountain View, CA, USA) was used for yeast two-hybrid (Y2H) screening. *E. coli* and *E. amylovora* strains were routinely grown at 37 °C and 28 °C, respectively, in Luria Bertani (LB) media with appropriate antibiotics. Yeast cells were cultured in YPD medium (1% w/v yeast extract, 2% w/v bacto peptone, 2% glucose) or grown in SD (synthetic defined) media with 2% glucose and lacking tryptophan and leucine. Antibiotics used included ampicillin (100 µg ml^−1^), kanamycin (50 µg ml^−1^) and chloramphenicol (50 µg ml^−1^).

### 4.4. Plasmid Construction

For growth curve and cell morphology analysis, putative toxins or entire TA loci were cloned into either pDEST527, pBAD33 [23] or pEVS143 [24] plasmids. A list of primers used in this study is provided in Table S3. For expression in pDEST527, toxin genes encoding CbtA, GNAT and xre-GNAT loci from CTBT3-1 were amplified using cbtAtopo F-R, GNATtopo F-R and XGtopo-F-GNATtopoR primers, respectively, and cloned into pENTR-D-Topo (Invitrogen, Carlbad, CA, USA) according to the manufacturer’s instruction, generating pENTR-D-topo*cbtA*, pENTR-D-topoGNAT and pENTR-D-topoXG, respectively. Topo clones were further recombined into Gateway compatible destination vector pDEST527 using LR Clonase II enzyme mix according to the manufacturers’ instructions (Invitrogen, Carlsbad, CA, USA) to yield pDEST-*cbtA*, pDEST-GNAT and pDEST-*xre*-GNAT, respectively. The plasmids were sequenced and then transformed into *E. coli* strain BL21 (DE3). For the construction of pBAD33 plasmids, toxin genes *parE*, *hicA*, *doc* and *vapC* and entire TA loci *rhh-parE* and *phd-doc* from CTBT3-1 were amplified using pbadparE F-R, pbadhic F-R, pbaddoc F-R and pbadvap F-R, pbadrhh F-pbadparE R and pbadphd F- pbaddoc R primers, respectively, then cloned into XbaI-HindIII sites of pBAD33 to generate pBAD-*parE*, pBAD-*hicA*, pBAD-*doc*, pBAD-*vapC*, pBAD-*rhh-parE* and pBAD-*phd-doc*, respectively. The plasmids were sequenced and then transformed into *E. coli* BL21 (DE3). For the construction of pEVS143 plasmids, toxin genes *cbtA*, *hicA* and *vapC* and the entire *cbeA-cbtA* locus from CTBT3-1 were amplified using primers pevscbtA F-R, pevshicA F-R, pevsvap F-R and pevscbeA F- pevscbtA R, then cloned into EcoRI- BamHI restriction sites of pEVS143 to generate pEVS-*cbtA*, pEVS-*hicA*, pEVS-*vapC* and pEVS-*cbeA-cbtA*, respectively. The plasmids were sequenced and transformed into *E. amylovora* CTBT3-1. The control vector pEVScv was constructed by cloning first 150 nt of locus EAMY_1412 into EcoRI-BamHI sites of pEVS143 using pEVScvF and R, respectively. For the construction of bait plasmids for Y2H assay, *cbeA* from CTBT3-1 and *cbtA* from strain BW25113 were amplified using cbeAtopo F-R and EccbtAtopoF-R and cloned into pENTR-D-topo using the manufacturer’s instructions. pENTR-D-topo*cbtA*, pENTR-D-topo*cbeA* and pENTR-D-topo*cbtAEc* were further recombined to the Gateway compatible bait plasmid pASGW-attR [25] using an LR Clonase reaction. For the construction of the prey plasmid, *mreB* and *ftsZ* were amplified from CTBT3-1 using mreBtopoEa F-R, ftsZtopoEa F-R; *ftsZ* was amplified from BW25113 using ftsZtopoEc F-R and cloned pENTR-D-topo according to the manufacturer’s instructions. pENTR-D-topo*mreB*, pENTR-topo*ftsZ* and pENTR-D-topo*cftsZEc* were recombined into Gateway compatible prey plasmid pACTGW-attR [25] as described above.

### 4.5. Monitoring Growth Rates of E. coli and E. amylovora Expressing Toxins or TA Loci

To determine the effect of toxin or TA loci expression on growth, cultures of *E. coli* strain BL21 (DE3) harboring pDEST527 plasmids were adjusted to 10^6^ CFU/mL (OD_600_ = 0.001) in LB media with or without 1 mM IPTG. For expression from pBAD33 plasmids, cultures were adjusted to 10^6^ CFU/mL (OD_600_ = 0.001) in LB media with or without 0.4% arabinose. Each culture was distributed into three wells of a 96-well plate. Cultures were grown in 200 μL volumes at 37 °C under continuous orbital shaking in a Synergy H2 microplate reader (Biotek, Winooski, VT, USA) and OD_600_ was measured every 20 min. For the analysis of *E. amylovora* CTBT3-1 harboring pEVS143 plasmids, cultures were adjusted to 10^6^ CFU/mL (OD_600_ = 0.001) in LB media with or without 1 mM IPTG and OD_600_ was measured every 45 min at 28 °C. For all assays, at least three independent experiments were performed.

### 4.6. Microscopic Visualization of Cell Morphology

Overnight cultures of *E. coli* strain BL21 (DE3) harboring pDEST527 plasmids and *E. amylovora* strain CTBT3-1 harboring pEVS143 plasmids were freshly diluted (1:100) in LB medium. At OD_600_ ~0.3, the expression of the cloned genes was induced by the addition of 1 mM IPTG. Cells were harvested 5 h post-inoculation (hpi), heat fixed and strained with crystal violet. Images were captured using a Zeiss Axio Microscope (Zeiss, Oberkochen, Germany).

### 4.7. Yeast Two-Hybrid Assays

The genes of interest were fused to the GAL4 DNA binding domain generating bait fusion constructs or the DNA activation domain generating prey fusion constructs. Bait and prey plasmid constructs were transformed into *Saccharomyces cerevisiae* strain Y2H gold with a Frozen-EZ Yeast Transformation Kit (Zymo Research, Irvine, CA, USA) according to the manufacturer’s instructions. The transformed cells were further plated onto SD (synthetic defined) media with glucose (2%) and lacking tryptophan and leucine or selection plates lacking histidine along with tryptophan and leucine. The plates were incubated at 30 °C for 3 days and the appearance of growth was recorded.

## Figures and Tables

**Table 1:** Putative type II/IV toxin–antitoxin (TA) systems predicted in *Erwinia amylovora* strain CTBT3-1 and the reference genome CFBP1430. Systems selected for functional analysis are in bold type.

No	Locus Tag (CFBP1430)	Length(aa)	Strand	Family/Domain	TAfinder	SLING
Chromosome						
1	EAMY_0399	107	+	**YeeU/CbeA (A)**	P	P
	EAMY_0400	106	+	**YeeV/CbtA (T)**		
2	EAMY_0402	82	+	**RHH (A)**	P	P
	EAMY_0403	91	+	**RelE/ParE (T)**		
3	EAMY_0675	222	-	DUF4258	NP	P
	EAMY_0676	100	-	-		
4	EAMY_1249	135	+	**Xre (A)**	P	NP
	EAMY_1250	137	+	**GNAT (T)**		
5 *	EAMY_1411	240	+	Pfam 00196 (A)	P (QS system)	NP
	EAMY_1412	207	+	COG03916 (T)		
6	EAMY_1772	73	+	**PhD (A)**	P	P
	EAMY_1773	123	+	**Fic/Doc(T)**		
7	EAMY_1828	59	+	**HicB (A)**	P	P
	EAMY_1829	136	+	**HicA (T)**		
8	EAMY_2645	144	-	RatA	NP	P
	EAMY_2644	95	-	-		
9	EAMY_2779	46	-	ParE (T)	P	NP
	EAMY_2780	75	-	RHH (A)		
Plasmid						
1	EAMY_3728	81	+	**PhD (A)**	P	P
	EAMY_3729	130	+	**VapC/PIN (T)**		
2	EAMY_3729	130	+	VapC (T)	P	NP
	EAMY_3730	57	+	Pfam 01527 (A)		

A: antitoxin; T: toxin; P: predicted; NP: not predicted; -: no domain/family found; *: identified as quorum sensing system.

## Supplementary Materials

The following are available online at https://www.mdpi.com/2072-6651/11/4/206/s1, File S1: TA systems predicted by SLING, Figure S1: Distribution of the number of TA systems in genomes of *E. amylovora* (*n* = 11), other *Erwinia* pathogens (P) (*n* = 6) and nonpathogens (NP) (*n* = 7), Table S1: Accession numbers of CbtA homologs used for phylogenetic analysis, Table S2: Strains and plasmids used in the study, Table S3: Primers used in the study.
