# Supplementary material for: Survey of Toxin–Antitoxin Systems in Erwinia amylovora Reveals Insights into Diversity and Functional Specificity
Source: Toxins (Basel). 2019 Apr 6;11(4):206. doi: 10.3390/toxins11040206 (PMC6521040; doi:10.3390/toxins11040206)
Supplement: Supplementary file 1 [file toxins-11-00206-s001.zip › Supplimentary material.pdf]

Supplementary Materials: Survey of Toxin–Antitoxin Systems in *Erwinia amylovora* Reveals Insights into Diversity and Functional Specificity

Teja Shidore, Quan Zeng and Lindsay R. Triplett

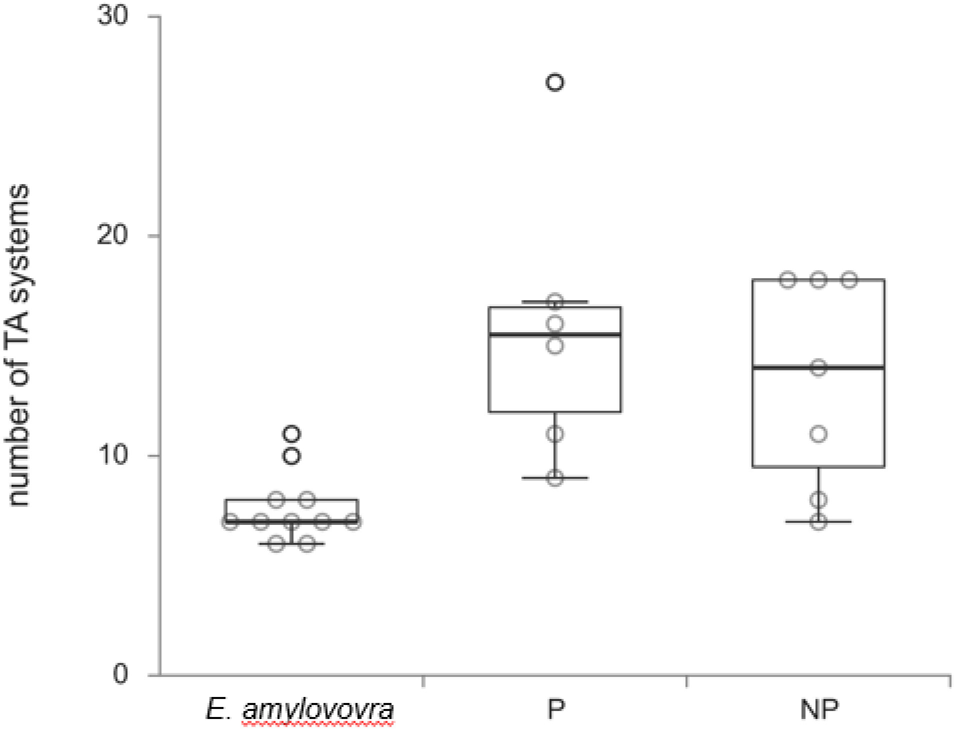

Figure S1. Average number of TA systems in genomes of *E. amylovora* (n = 11), other *Erwinia* pathogens (P) (n = 6) and nonpathogens (NP) (n = 7).

Table S1. Accession numbers of strains used in CbtA phylogenetic analysis.

No	Strain	Accession No
1	*E. amylovora* LA092	WP_099319755
2	*E. amylovora* MAGFLFL 2	WP_004155189.1
3	*E. amylovora* 01SFRBO	CCO88626.1
4	*E. amylovora* ATCC 49946	CBJ47694.1
5	*E. amylovora* CFBP1430	CBA19342.1
6	*E. amylovora* CTBT3-1	WP_004155189.1
7	*E. amylovora* UPN527	CCO97736.1
8	*E. amylovora* E-2	WP_004155189.1
9	*E. amylovora* NHSB01-1	WP_004155189.1
10	*E. amylovora* CTBT3-1	WP_004155189.1
11	*E. amylovora* MASHBO	WP_004155189.1
12	*E. oleae* DAPP-PG531	WP_034949502.1
13	*P. ananatis* LMG 5342	CCF08502.1
14	*P. parmentieri* WPP163	ACX87181.1

15	*P. atrosepticum* SCRI1043	CAG75753.1
16	*P. carotovorum* PCC21	AFR04662.1
17	*D. zeae Ech586*	ACZ75307.1
18	*E. coli* W_1	WP_001095907.1
19	*E. coli* W_2	WP_000691790.1
20	*E. coli* BW25113_1	AIN30763.1
21	*E. coli* BW25113_2	AIN33039.1
22	*E. coli* BW25113_3	AIN32423.1
23	*E. coli* CFT073_1	AAN80986.1
24	*E. coli* CFT073_2	AAN78757.1
25	*E. coli* CFT073_3	AAN82125.1
26	*E. coli* CFT073_4	AAN79761.1
27	*E. coli* CFT073_5	AAN83010.1
28	*E. coli* CFT073_6	AAN83571.1
29	*E. coli* O157:H7 Sakai_1	WP_000854914.1
30	*E. coli* O157:H7 Sakai_2	WP_000854712.1
31	*E. coli* O157:H7 Sakai_3	WP_001303564.1

Table S2. Strains and plasmids used in the study.

Strains and Plasmids	Relevant Characters	Source
	Escherichia coli	
DH5 *α*	F - 80dlacZ ΔM15 Δ(lacZYA-argF)U169 endA1 recA1 hsdR17(r K - m K +) deoR thi-1 supE44 gyrA96 relA1 *λ*	Invitrogen
BL21(DE3)	fhuA2 ompT gal (*λ* DE3) (dcm) ΔhsdS *λ* DE3 = *λ* sBamHIo ΔEcoRI-B int::(lacI::PlacUV5::T7 gene1) i21 Δnin5	Coli Genetic Stock Center (CGSC)
BW25113	*$lacI^{q}$ $rrnB_{T14}$ $\Delta lacZ_{WJ16}$ hsdR514 $\Delta araBAD_{AH33}$ $\Delta rhaBAD_{LD78}$*	CGSC
	Erwinia amylovora	
CTBT3-1	Wild type	[3]
	Plasmids	
pENTR-D-Topo	Entry vector for Gateway cloning technology, Km^R	Invitrogen
pENTR-D-topo*cbtA*	*cbtA* from CTBT3-1 cloned into pENTR-D-topo, Km^R	This study
pENTR-D-topo*cbeA*	*cbeA* from CTBT3-1 cloned into pENTR-D-topo, Km^R	This study
pENTR-D-topoGNAT	Gene encoding GNAT protein from CTBT3-1 cloned into pENTR-D-topo, Km^R	This study
pENTR-D-topoXG	*xre-GNAT* loci from CTBT3-1 cloned into pENTR-D-topo, Km^R	This study
pENTR-D-topo*cbtAEc*	*cbtA* from *E. coli* strain BW25113 cloned into pENTR-D-topo, Km^R	This study
pENTR-D-Topo*ftsZEc*	*ftsZ* from E. coli strain BW25113 cloned into pENTR/D-topo, Km^R	This study
pENTR-D-topo*mreB*	*mreB* from CTBT3-1 cloned into pENTR-D-topo, Km^R	This study
pENTR-D-topo*ftsZ*	*ftsZ* from CTBT3-1 cloned into pENTR-D-topo, Km^R	This study
pDEST-527	Gateway destination vector for *E. coli* expression, T7 promoter, N-terminal 6xHis tag, Amp^R, Cm^R	Addgene.org
pDEST-*cbtA*	insert from pENTR-D-topo*cbta* recombined into pDEST-527, Amp^R	This study
pDEST-GNAT	insert from pENTR-D-topoGNAT recombined into pDEST-527	This study
pDEST-xre-GNAT	insert from pENTR-D-topoXG recombined into pDEST-527, Amp^R	This study
pBAD33	Expression vector allowing cloning of target genes downstream of the P_{BAD} promoter, Cm^r	[23]

pBAD-*parE*	XbaI-HindIII flanked *parE* from CTBT3-1 cloned in pbad33, Cm^r	This study
pBAD-*rhh-parE*	XbaI-HindIII flanked *rhh-parE* loci from CTBT3-1 cloned in pbad33, Cm^r	This study
pBAD-*doc*	XbaI-HindIII flanked *doc* from CTBT3-1 cloned in pbad33, Cm^r	This study
pBAD-*phd-doc*	XbaI-HindIII flanked *phd-doc* loci from CTBT3-1 cloned in pbad33	This study
pBAD-*hicA*	XbaI-HindIII flanked *hicA* from CTBT3-1 cloned in pbad33, Cm^r	This study
pBAD-*vapC*	Xba-HindIII flanked *vapC* from pEA29 plasmid cloned in pbad33, Cm^r	This study
pEVS143	Broad-host-range cloning vector; inducible Cm^r and GFP; Km^r	[24]
pEVScv	150 bp of EAMY 1412 cloned into EcoRI-BamHI sites of pesv143, Km^r	This study
pEVS-*cbtA*	EcoRI-BamHI flanked *cbtA* from CTBT3-1 cloned into pesv143, Km^r	This study
pEVS-*cbeA-cbtA*	EcoRI-BamHI flanked *cbeA-cbtA* loci from *CTBT3-1* cloned into pesv143, Km^r	This study
pEVS-*hicA*	EcoRI-BamHI flanked *hicA* from CTBT3-1 cloned into pesv143, Km^r	This study
pEVS-*vapC*	EcoRI-BamHI flanked *vapC* from pEA29 plasmid cloned into pesv143, Km^r	This study
pACTGW-attR	Gateway destination vector with Gal4-AD domain, Amp^R	[25]
pASGW-attR	Gateway destination vector with Gal4-BD domain, Amp^R	[25]
pACTGW-attR*cbtA*	Insert from pENTR/D-topo*cbtA* recombined into pACTGW-attR, Amp^R	This study
pACTGW-attR*cbeA*	Insert from pENTR/D-topo*cbeA* recombined into pACTGW-attR, Amp^R	This study
pACTGW-attR*mreB*	Insert from pENTR/D-topo*mreB* recombined into pACTGW-attR, Amp^R	This study
pACTGW-attR*ftsZ*	Insert from pENTR/D-topo*ftsZ* recombined into pACTGW-attR, Amp^R	This study
pACTGW-attR*cbtAEc*	insert from pENTR/D-topo*cbtAEc* recombined into pACTGW-attR, Amp^R	This study
pACTGW-attR*ftsZEc*	insert from pENTR/D-topo*ftsZEc* recombined into pACTGW-attR, Amp^R	This study
pASGW-attR*cbtA*	Insert from pENTR/D-topo*cbtA* recombined into pASGW-attR, Amp^R	This study
pASGW-attR*cbeA*	Insert from pENTR/D-topo*cbeA* recombined into pASGW-attR, Amp^R	This study
pASGW-attR*mreB*	Insert from pENTR/D-topo*mreB* recombined into pASGW-attR, Amp^R	This study
pASGW-attR*ftsZ*	Insert from pENTR/D-topo*ftsZ* recombined into pASGW-attR, Amp^R	This study
pASGW-attR*cbtAEc*	insert from pENTR/D-topo-*cbtAEc* recombined into pASGW-attR, Amp^R	This study
pASGW-attR*ftsZEc*	insert from pENTR/D-topo-*ftsZEc* recombined into pASGW-attR, Amp^R	This study

Table S3. Primers used in the study.

Primer	Sequence 5′-3′
cbtAtopo F	caccatgcacatttcaactgtaccg

cbtAtopo R	ttaaatatttattaatccggtagctcg
GNATtopoF	caccatgacggactatcaatgg
GNATtopoR	ttagagcgattcagcgatatc
XGtopoF	caccctgaggctggaaaaaaactgg
EccbtAtopo F	caccatgaaaacattacctgtattacccg
EccbtAtopo R	tcatttcgcctccggatactta
pBADparEF	gatctctagaaggaggagagtaatgagctatgaactgctcttcg
pBADparER	gatcaagctt tcaaacgcttataaccaacatt
pBADrhhF	gatctctagaaggaggagagtaatgagacaactcgcgaaacaga
pBADhicF	gatctctagaaggaggagagtaatgatagaggccgatggat
pBADhicR	gatcaagctt ttaaagccccgcctgttt
pBADdocF	gatctctagaaggaggagagtaatgacggactatcaatgg
pBADdocR	gatcaagctt ttagagcgattcagcgatatc
pBADphdF	gatctctagaaggaggagagtaatgcaagctataaactttactaccg
pBADvapF	gatctctagaaggaggagagtaatgttcatggcctatgagccagaaa
pBADvapR	gatcaagcttctaccggggcgggggcagt
pbadrhhF	gatctctagaaggaggagagtaatgagacaactcgcgaaacaga
pbadphdF	gatctctagaaggaggagagtaatgcaagctataaactttactaccg
pEVScbtA F	gatcgaattcatgcacatttcaactgtaccg
pEVScbtA R	gatcggatccttaaatatttattaatccggtagctcg
pEVShic F	gatcgaattcatgatagaggccgatggatg
pEVShic R	gatcggatccttaaagccccgcctgttttt
pEVSvap F	gatcgaattcatgttcatggcctatgagccagaaa
pEVSvap R	gatcggatccctaccggggcgggggcagt
pEVScbeA F	gatcgaattcatgtcaatcacactacctca
pEVScvF	gatcgaattcatgaaaatcattcagaccaa
pEVScvR	gatcggatccatcgtaacgatcgtactcc
cbeAtopoF	caccatgtcaatcacactacctca
cbeAtopoR	ttatggggttggataaatag
mreBtopoEaF	caccatgtttaaaaaattccgaggc
mreBtopoEaR	ttattcttcgctgaacaaatc
ftsZtopoEaF	caccatgtttgaaccaatggaactaa
ftsZBtopoEaR	ttaatctgcctgcttacgc
ftsZtopoEcF	caccatgtttgaaccaatggaactta
ftsZtopoEcF	ttaatcagcttgcttacgcagg
EccbtAtopoF	caccatgaaaacattacctgtattacccg
EccbtAtopoR	tcatttcgcctccggatactta
